# Computed Tomographic and Magnetic Resonance Imaging Diagnosis of Concurrent Sinonasal Aspergillosis and Meningoencephalocele in a Dog

**DOI:** 10.1155/crve/6620660

**Published:** 2025-04-22

**Authors:** Grant Ford-Hodges, Silke Hecht, Jacqueline C. Whittemore

**Affiliations:** Department of Small Animal Clinical Sciences, University of Tennessee College of Veterinary Medicine, Knoxville, Tennessee, USA

**Keywords:** CT, fungal rhinitis, MRI, rhinoscopy, seizure

## Abstract

A 4-year-old male castrated mixed breed dog was admitted to the Veterinary Medical Center of the University of Tennessee College of Veterinary Medicine for evaluation of unilateral nasal discharge. Discharge had been present for 2 months, with progression from purulent to hemorrhagic discharge after empiric treatment by the primary veterinarian 6 weeks prior to referral. The dog had a history of seizures starting at 1 year of age that were controlled with phenobarbital. Computed tomography and magnetic resonance imaging of the head yielded diagnoses of a left-sided meningoencephalocele with extension of the olfactory bulb into the caudal nasal passage and destructive rhinitis and frontal sinusitis consistent with aspergillosis. Rhinoscopy was performed to collect diagnostic samples, debride fungal plaques, and guide clotrimazole cream application. Biopsies revealed suppurative rhinitis with abundant aspergillosis, with *Aspergillus* sp. confirmed on fungal culture. Postoperatively, the patient was treated with a short course of oral posaconazole. Stertorous breathing was noted 4 months following treatment. Recheck sinoscopy and rhinoscopy revealed one fungal plaque in the left nasal cavity and small, hyperemic turbinates. Treatment was repeated, resulting in resolution of clinical signs. Repeat rhinoscopy 1 month later had no evidence of residual disease. Seizures recurred 3 years after the second treatment and were medically managed by the primary care veterinarian. This case report describes a rare case of nasal aspergillosis complicated by a meningoencephalocele. Despite the large cribriform plate defect resulting in exposure of the meninges and olfactory bulb, repeated debridement and topical antifungal treatment were well tolerated by this patient. There was no recurrence in signs for 3 years, after which the dog was lost to follow-up.

## 1. Introduction

Nasal disease is a common presenting complaint for dogs in small animal practice [1]. Possible etiologies include rhinitis, neoplasia, sinonasal aspergillosis, nasal foreign bodies, periodontal disease, and others [2, 3]. While a presumptive diagnosis can sometimes be established based on a patient's signalment, history, and physical examination findings, diagnostic imaging is typically employed in the diagnostic workup, followed by rhinoscopy. Nasal radiography is inferior to cross-sectional imaging and is no longer commonly used [4]. Computed tomography (CT) is the preferred imaging test for the evaluation of the small animal nasal cavity and paranasal sinuses due to the speed of image acquisition, the ability to acquire submillimeter slices and generate multiplanar reconstructed (dorsal, sagittal, and oblique) images, and the excellent resolution of both osseous structures and air-filled spaces [5, 6]. Magnetic resonance imaging (MRI) is the gold standard for the evaluation of intracranial disease processes. Based on available literature, it also allows adequate assessment of nasal disease in dogs [7, 8]. However, CT is usually given preference due to the above advantages and typically lower cost to the client [6].

Sinonasal aspergillosis in dogs most often is caused by *Aspergillus fumigatus*, although infection with other species is possible [1, 9]. Medium to large, dolichocephalic or mesaticephalic dogs are predisposed. There is no true age predilection, but many animals present at young to middle age [10]. Clinical signs at presentation include chronic serous, mucopurulent or sanguinopurulent nasal discharge and episodic epistaxis which is often unilateral, especially in the earlier stages of the disease. Additional abnormalities may include pain, facial deformity, regional depigmentation, or ocular involvement [9]. CT findings with sinonasal aspergillosis are fairly characteristic and well described in the literature and include turbinate lysis, mucosal thickening, and hyperostosis of the nasal cavity and the frontal sinus [11–13]. In addition to imaging, the diagnosis of sinonasal aspergillosis hinges on rhinoscopy/sinoscopy, histology, and fungal culture [10]. Sinonasal aspergillosis can be treated by infusion of enilconazole or clotrimazole solution using endoscopically placed frontal sinus catheters or nasal catheters [14, 15]. Historically, topical treatment has been considered contraindicated in cases with cribriform or sinus compromise, due to the potential for refractory seizures [16]. For this reason, assessment of the integrity of the cribriform plate is an important part of the image interpretation in patients with suspected sinonasal aspergillosis.

Meningocele (herniation of meninges) and meningoencephalocele (herniation of cerebral tissue along with meninges) through calvarial defects are uncommon in dogs, with only few isolated case reports and one larger case series found in the literature [17–23]. While a superficial/parietal defect may be identified on physical examination, the diagnosis of a deep (nasal and ethmoidal) meningocele/meningoencephalocele requires cross-sectional imaging. With this condition, meninges +/− brain parenchyma herniate through a cribriform plate defect into the caudal nasal cavity. The most commonly reported clinical signs are seizures or behavior changes. Other abnormalities including facial deformities are possible. Both nonsurgical and surgical treatment approaches have been described, but medical management appears to be the preferred approach at this point [19].

Little information is available in the literature regarding concurrent meningoencephalocele and acquired nasal disease. One case report describes acute worsening of seizures due to suppurative meningoencephalitis secondary to suppurative rhinitis and infection of the meningoencephalocele [22]. To the authors' knowledge, only one other case of concurrent aspergillosis and nasal meningoencephalocele in a dog has previously been reported [20]. In that case, aspergillosis was diagnosed approximately 5 months following transfrontal craniotomy to resect the meningoencephalocele protrusion and close the dural defect. This case report describes a case of sinonasal aspergillosis in a dog complicated by a meningoencephalocele which was successfully managed medically. Despite a large cribriform plate defect resulting in exposure of the meninges and olfactory bulb, repeated debridement and topical antifungal treatment were well tolerated. There was no recurrence in signs for 3 years, after which the dog was lost to follow-up.

## 2. Case Presentation

A 4-year-old male castrated mixed breed dog was admitted to the Veterinary Medical Center of the University of Tennessee College of Veterinary Medicine for diagnostic work-up of chronic epistaxis. Discharge had been noted 2 months previously, at which time it was purulent. The dog was evaluated by the primary care veterinarian at that time. A complete blood count was unremarkable. The dog was treated empirically with amoxicillin clavulanate (12.3 mg/kg, PO, q 12 h), diphenhydramine (1.97 mg/kg, PO, q 8–12 h), and saline nasal drops (q 8–12 h) by the primary care veterinarian. The dog also had a history of seizures, diagnosed 3 years prior (at 1 year of age). Diagnostic imaging of the head was not performed at that time. Toxic and metabolic causes were ruled out, after which the primary care veterinarian made a presumptive diagnosis of idiopathic epilepsy and prescribed phenobarbital (2.4 mg/kg, PO, q 12 h). Seizures had remained well controlled since starting phenobarbital.

The dog's nasal discharge progressed to epistaxis over the course of 6 weeks despite completion of the previously prescribed therapies. Appetite, water consumption, bowel movements, and urination remained normal. Sedated oral examination performed by the primary veterinarian revealed a single 1-cm nonpigmented, nonulcerated, and nonpainful area on the hard palate of the dog's mouth, mesial to his left canine. There was no apparent swelling in the oral cavity or muzzle. Referral to the authors' institution was recommended.

On physical examination performed at the authors' institution, the dog had epistaxis from the left nostril, depigmentation of that nare, and mild enlargement of the mandibular lymph nodes. The dog was unwilling to open his mouth due to apparent pain. Clinicopathologic abnormalities included macrocytic, hypochromic anemia (hematocrit 34.8%, reference range (RR) 41–60%; mean cell volume 76.7 fL, RR 62–74 fL; mean corpuscular hemoglobin concentration 33.4 g/dL, RR 34.5–36.3 g/dL), leukocytosis (15,600 WBCs/*μ*L, RR 5100–14,000 WBCs/*μ*L; segmented neutrophils 12,450/*μ*L, RR 2.65–9.8/*μ*L), hypocalcemia (8.9 mg/dL, RR 10–12 mg/dL), decreased total protein (5.0 g/dL, RR 5.4–6.8 g/dL), and hypoalbuminemia (2.1 g/dL, RR 3.2–4.3 g/dL). Prothrombin time (PT) and partial thromboplastin time (PTT) were unremarkable. Thoracic radiographs were normal.

**Figure 1:**
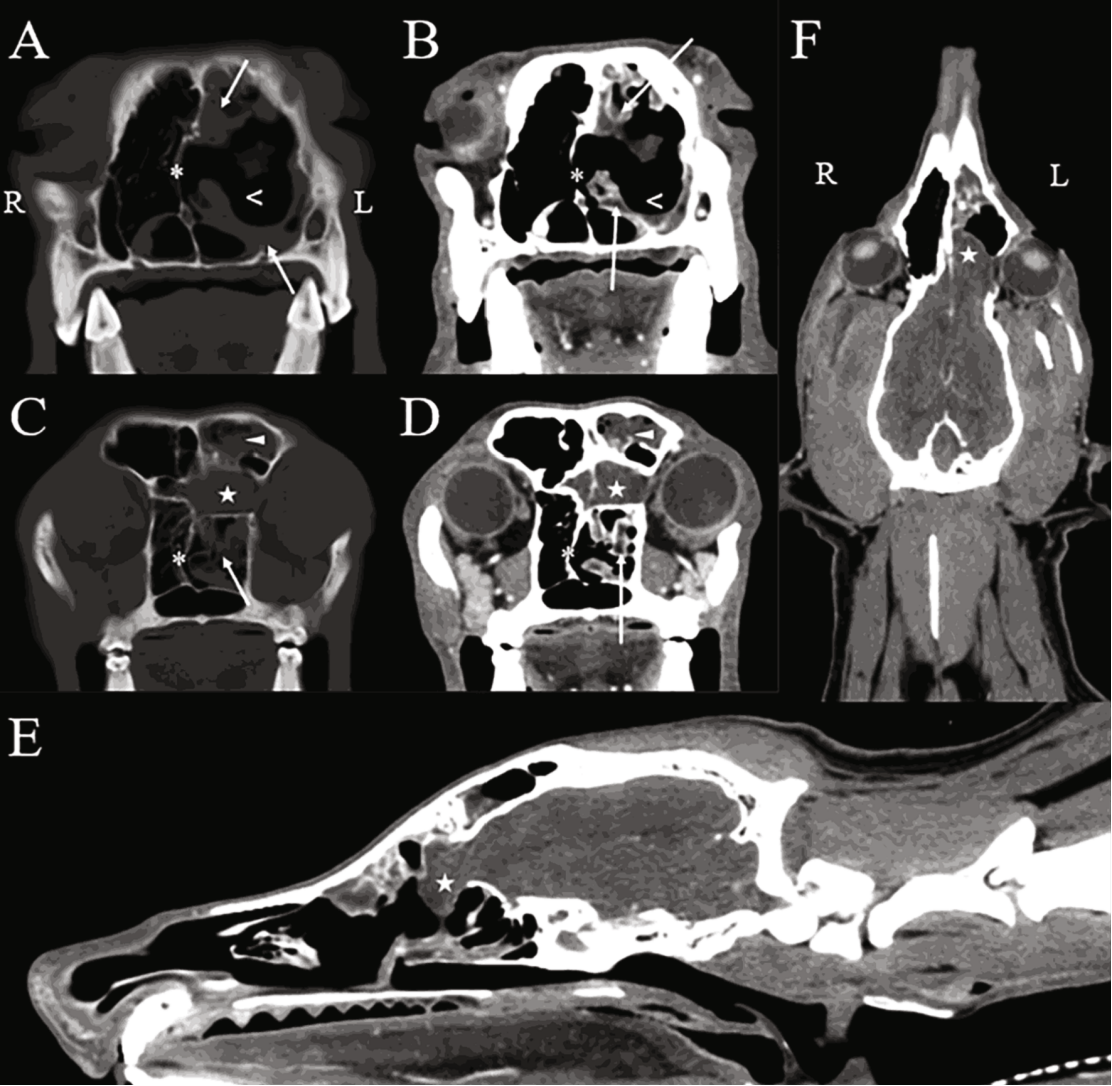
Precontrast bone algorithm (A, C) and postcontrast soft tissue algorithm (B, D) reconstructed head CT images. Transverse plane images of the nasal cavity (A–D) at the level of the rostral nasal cavity (A, B) and frontal sinus (C, D). Postcontrast sagittal (E) and dorsal (F) plane reconstructed images from the nares to the cranial cervical spine. There is marked rightward deviation of the nasal septum (asterisks (⁣^∗^); A–D) and marked lysis of the nasal and ethmoid turbinates (open arrowheads; A, B) on the left side. A moderate amount of fluid attenuating, noncontrast enhancing material surrounds the residual turbinates (arrows; A–D). Additionally, a moderate amount of noncontrast enhancing fluid attenuating material is identified within the left frontal sinus (arrowhead; C, D). A large defect within the left part of the cribriform plate and extending to the rostral aspect of the orbital part of the frontal bone is evident on both sagittal and dorsal plane images with extension of the left olfactory bulb through the cribriform defect into the caudal aspect of the nasal passage (star; C–E). There is mild contrast enhancement of the meninges. R = right; L = left.

**Figure 2:**
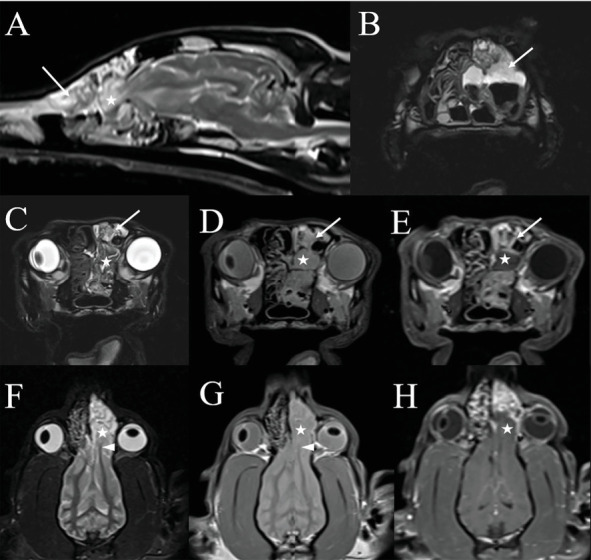
MRI study of the head. Left parasagittal T2-weighted image (A); transverse thin section 3D T2-weighted images at the level of the mid nasal cavity (B) and cribriform plate (C); transverse PD-weighted (D) and postcontrast T1-weighted (E) images at the level of the cribriform plate; and dorsal plane STIR (F), PD-weighted (G), and postcontrast T1-weighted with fat saturation (H) images. (B–D) There is again evidence of extensive turbinate destruction within the left nasal cavity, with T2 hyperintense material distributed between residual turbinates (arrows). (A, C–H) The large left cribriform plate defect with herniation of the olfactory bulb (star) is evident on both sagittal and dorsal plane images with extension of the left olfactory bulb through the cribriform defect into the caudal aspect of the nasal passage (star). Especially on dorsal plane STIR (F) and PD-weighted (G) images, there is asymmetry of the olfactory bulbs, with rostral extension and a stretched appearance of the left rostral white matter tracts compared to the right (solid arrowhead). (H) Moderate contrast enhancement of the meninges is evident adjacent to the herniated olfactory bulb.

CT and MRI of the head were performed 1 day apart (Figures 1 and 2). The dog was anesthetized and placed in sternal recumbency for both procedures. Premedication for each procedure consisted of butorphanol (0.4 mg/kg, IV), dexmedetomidine (1.2 *μ*g/kg, IV), and maropitant (1 mg/kg, IV). The dog was induced with propofol (6 mg/kg, IV) and midazolam (0.3 mg/kg, IV), and it then was maintained on isoflurane inhalant anesthesia (1.0–2.0%). The dog was placed in sternal recumbency for CT of the head using a 40-slice helical CT scanner (Philips Brilliance-40, Philips International B.V., Amsterdam, Netherlands). The images were acquired in a transverse plane with helical acquisition mode (120 kV, 237 mA, slice thickness = 0.9 mm and 4 mm, matrix = 512 × 512, and pitch 0.5) and were reconstructed using the bone and soft tissue algorithms. A bone window (preset window center 600 HU, window width 2600 HU) and soft tissue window (preset window center 50 HU and window width 350 HU) were used for evaluation. Positive contrast medium (Ioversol; Optiray 350 mgI/mL, Guerbet LLC, Princeton, NJ) at 1 mL/lb was injected through the cephalic vein. Multiplanar reconstructed images in the sagittal and dorsal planes were generated from the original transverse image datasets for further evaluation.

The CT images (Figure 1) revealed malformation of the osseous structures of the cranial vault characterized by a malformed left frontal bone and maxilla causing dorsoventral narrowing and compartmentalization of the frontal sinus. A large defect (1.8 cm in width by 1 cm in height) was seen within the left part of the cribriform plate that extended to involve the rostral aspect of the orbital part of the frontal bone. A small defect also was noted within the right part of the cribriform plate (0.5 cm in width by 0.1 cm in height). There was an extension of the left olfactory bulb through the cribriform defect into the caudal aspect of the nasal passage, and mild contrast enhancement of the meninges was seen at this level. There was rightward deviation of the nasal septum and marked lysis of the nasal and ethmoid turbinates within the left nasal cavity. A moderate amount of fluid attenuating, noncontrast enhancing material surrounded the residual turbinates, and mild thickening of the mucosal lining was identified following contrast medium administration. A moderate amount of noncontrast enhancing fluid attenuating material also was identified within the left frontal sinus. The osseous boundaries of the left nasal cavity and frontal sinus were mildly, irregularly thickened. Additional abnormalities not shown in the selected figures included mild to moderate enlargement of the left mandibular lymph nodes and both medial retropharyngeal lymph nodes.

The CT findings were consistent with a left-sided meningoencephalocele with extension of the olfactory bulb into the caudal nasal passage. Concurrent findings of ipsilateral destructive rhinitis and frontal sinusitis were most consistent with an infectious process (specifically, aspergillosis) [11–13]. Secondary meningitis was suspected based on contrast enhancement of the meninges covering the olfactory bulb.

Per recommendations from the attending neurologist, an MRI examination and cisternal CSF tap with cytology were performed in concert with rhinoscopy to evaluate for meningitis and intracranial parenchymal disease that might alter recommendations regarding systemic antifungal and antiepileptic therapy. The MRI study was performed using a 1.5 Tesla superconducting MRI system (MAGNETOM Espree, Siemens Medical Solutions, Malvern, PA) equipped with a head coil and consisted of T2-weighted images in the sagittal and transverse planes (TR 4230–4510 ms and TE 101 ms); T1-weighted images in the transverse plane (TR 527 ms and TE 12 ms); proton density weighted images in the dorsal, sagittal, and transverse planes (TR 2730 ms and TE 13 ms); a fluid attenuated inversion recovery (FLAIR) sequence in the transverse plane (TR 9820 ms, TE 75 ms, and TI 2588 ms); a T2⁣^∗^-weighted gradient recalled echo (GRE) sequence in the transverse plane (TR 1570 ms, TE 26 ms, and flip angle 20); a thin-section 3D T2 weighted (“SPACE”) sequence in the transverse plane (TR 1250 ms and TE 123 ms); and a short tau inversion recovery (STIR) sequence in the dorsal plane (TR 3540 ms, TE 40 ms, and TI 160 ms). Postcontrast sequences acquired following the intravenous administration of contrast medium (0.1 mmol/kg Magnevist; gadopentetate dimeglumine; Bayer Healthcare Pharmaceuticals Inc., Wayne, NJ) included T1-weighted images in the sagittal and transverse planes (TR 344–527 ms and TE 12 ms), T1-weighted images with fat saturation in the dorsal plane (TR 389 ms and TE 12 ms), and 3D thin-section T1-weighted GRE (“VIBE”) images in the transverse plane (TR 5.32 ms, TE 2.39 ms, and flip angle 10). Slices were 3 mm in thickness with an interslice gap of 10–20%, except for 3D sequences where slices were 1.5 mm in thickness. The MRI study was compared to the CT images; the malformations of the left frontal bone and maxilla, multifocal defects in the cribriform plate, and herniation of the olfactory bulb into the left nasal cavity again were seen. Especially on dorsal plane images, there was asymmetry of the olfactory bulbs, with rostral extension and a stretched appearance of the left rostral white matter tracts compared to the right (Figure 2). At that level, there was moderate contrast enhancement of the meninges. The nasal septum remained deviated to the right with unchanged marked lysis of the left nasal turbinates. The remaining nasal turbinates were thickened and markedly contrast enhancing. A moderate amount of fluid was present in the left frontal sinus and nasal cavity with a small amount in the ventral aspect of the right nasal cavity. The lateral ventricles of the brain were mildly asymmetric and mildly enlarged rostrally. Cytologic evaluation of CSF collected via cisternal tap was unremarkable.

**Figure 3:**
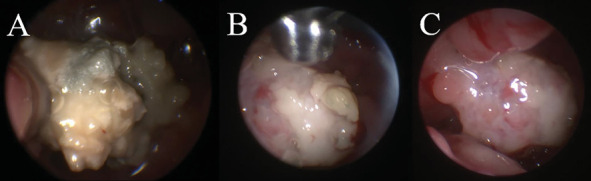
Rhinoscopic images of a large, white spongiform plaque (A) adhered to the herniated left olfactory bulb in a 4-year-old mixed breed dog with aspergillosis and a meningoencephalocele. The plaque was debrided using rhinoscopic instruments (B) to reveal underlying soft tissue (C).

Bilateral sinusotomy was performed to allow sinoscopic evaluation using a 2.7-mm rigid telescope. On sinoscopy, the left frontal sinus contained a small amount of fluid and thickened mucosa with fluid and gas-filled bubbles present rostrally. Plaques were not seen. The right frontal sinus was normal. The telescope then was advanced antegrade into the nose. Antegrade rhinoscopy revealed large, sparkling white plaques with a spongy surface, characteristic of *Aspergillus* sp., in the left lateral sinus adjacent to the orbit. Additionally, plaques were identified adhered to herniated olfactory bulb tissue (Figure 3). Plaques were collected for histopathology and culture from the lateral sinus and surface of the olfactory bulb. Endoscopic debridement of plaques and copious lavage with 0.9% saline were performed until all gross disease had been removed from the nose. Patency of the nasofrontal ostia was confirmed via identification of lavage fluid in the sinuses. The frontal sinuses then were packed with 1% clotrimazole cream using a 5–French red rubber catheter, with sinusotomy sites left open to heal by second intention. The dog was given a prophylactic dose of levetiracetam (60 mg/kg, IV, over 15 min, once) intraoperatively.

Anesthetic recovery was uneventful. The dog was discharged 1 day following his procedure pending biopsy and culture results. The dog was treated with clindamycin (20 mg/kg, PO, q 12 h for 7 days), posaconazole (5 mg/kg, PO, q 12 h for 7 days), levetiracetam (30 mg/kg, PO, q 12 h for 3 days), carprofen (2.2 mg/kg, PO, q 12 h for 14 days), gabapentin (10 mg/kg, PO, q 8–12 h for 7 days), and phenobarbital as previously prescribed by his primary care veterinarian.

Hematoxylin and eosin–stained sections of collected biopsies contained tangled mats of 5–10 *μ*m fungal hyphae with parallel walls, acute angle branching, and septation; numerous conidial heads; and aggregates of neutrophils, macrophages, and hemorrhage. Fungal culture grew *Aspergillus* sp. reported as consistent with *Aspergillus fumigatus* by the laboratory.

Progressive stertorous breathing was noted 4 months following hospitalization. Sinoscopy and antegrade rhinoscopy were repeated. The sinuses were unremarkable. One fungal plaque was identified in the left nasal cavity, along with multiple malformed hyperemic turbinates. The plaque was removed and aggressive lavage of the nose with 0.9% saline repeated, followed by 1% clotrimazole cream packing of the frontal sinuses. The dog was discharged with gabapentin (10 mg/kg, PO, q 8 h for 10 days), carprofen (2.2 mg/kg, PO, q 12 h for 5 days), levetiracetam (30 mg/kg, PO, q 12 h for 7 days), and phenobarbital as previously prescribed.

Stertorous breathing resolved after discharge, with no recurrence in respiratory signs on recheck 1 month later. Physical examination also was unremarkable. Repeat rhinoscopy did not reveal evidence of plaque recurrence. The dog did well at home following this appointment and had no recurrence in nasal or respiratory signs. The dog was presented to the primary care veterinarian with recurrence of seizures 3 years after referral. No neurologic abnormalities were identified on examination, and the primary care veterinarian recommended to increase the phenobarbital dose by 25–50%. The dog remained in the care of the primary care veterinarian during this time and then was lost to follow-up.

## 3. Discussion

Prior to referral and diagnostic imaging, the differential diagnoses that were considered in this dog included inflammatory/infectious rhinitis (including aspergillosis and bacterial rhinitis, even though the latter was considered less likely due to unilateral disease), foreign body, polyposis, and neoplasia. The mucosal depigmentation and imaging findings of cavitary destruction of the nasal turbinates with abnormal noncontrast enhancing mucinous tissue in the nasal passages, nasal and frontal sinus mucosal thickening, and reactive bony thickening were most consistent with nasal aspergillosis [11–13]. The diagnosis was supported by the characteristic appearance of plaques visualized with rhinoscopy and ultimately confirmed by histopathological evaluation and fungal culture.

The additional imaging abnormalities were unexpected and consistent with a deep/nasal/ethmoidal meningoencephalocele, defined as a herniation of cerebral tissue and meninges through a defect in the cranium from either a congenital incomplete closure of the calvarium or, less likely, acquired secondary to skull fractures [19]. A congenital abnormality was prioritized in this case because there was no history of trauma and no fractures were identified on imaging. Meningoencephaloceles in dogs are rare. The most common presenting clinical signs are epileptic seizures and abnormal behavior including aggressiveness, compulsive behavior, hyperactivity, intermittent yelping, and star-gazing [19]. Similarly, seizures are a common presenting complaint in people diagnosed with meningoencephaloceles [24]. The age of onset of seizures in dogs with meningoencephaloceles is variable, ranging from 1 month to 8 years [17–23].

Seizures can arise due to metabolic disease, toxicity, or intracranial disease. In our patient, seizures were presumed to be secondary to the meningoencephalocele given the dog's young age when they first manifested, lack of evidence of metabolic disease (such as hepatic encephalopathy), lack of historical trauma, lack of meningitis based on CSF tap, and lack of evidence of encephalitis or other lesions on CT and MRI studies. The dog's seizures had been well controlled with phenobarbital (2.4 mg/kg, PO, q 12 h) for 3 years at the time of presentation, which would be unexpected if they were due to infection.

Due to the infrequent occurrence of meningoencephaloceles in dogs, there are no accepted guidelines regarding treatment methods. Medical management using a combination of antiepileptics, anti-inflammatory doses of prednisolone, and/or antibiotics yielded adequate treatment response in most cases in one study [19]. To the authors' knowledge, only one case of concurrent aspergillosis and nasal meningoencephalocele in a dog has previously been reported [20]. That dog also had a history of epileptic seizures. In that case, aspergillosis was diagnosed approximately 5 months following transfrontal craniotomy to resect the meningoencephalocele protrusion and close the dural defect.

Sinonasal aspergillosis is a complex disease involving mucosal colonization with *Aspergillus* sp., fungal toxin release, and aberrant host response. Frontal sinus involvement is present in 75% of cases [25]. Regional depigmentation, as noted in the mouth by the primary veterinarian in this case, can raise suspicion of the disease in dogs with compatible clinical signs. Conversely, findings on routine laboratory testing are generally nonspecific and the presence of *Aspergillus* sp. on nasal swabs can be seen in dogs with rhinitis due to other causes. Diagnosis hinges on advanced imaging (such as CT), rhinoscopy/sinoscopy, histology, and fungal cultures [10]. Sinonasal aspergillosis can be treated by infusion of enilconazole or clotrimazole solution using endoscopically placed frontal sinus catheters or nasal catheters [14, 15]. Use of clotrimazole cream packed into the frontal sinuses has a comparable success rate to previous techniques [14], though up to four treatments can be required to achieve cure [26]. Clotrimazole cream placed in the sinuses has been reported to be retained as long as 96 h [27]. If cream is used, both the lateral and rostral compartments of the frontal sinuses should be packed [27], as was performed in this case.

Regardless of whether topical solution or cream is used, successful long-term outcome hinges on aggressive debridement of all gross fungal plaques and adequate azole concentration into the frontal sinuses. It consistently predicts the likelihood of success with a single treatment [28]. Regardless of whether solution, cream, or a combination is used, patients should undergo repeat rhinoscopy/sinoscopy every 2–3 weeks for detection and treatment of residual disease before meaningful regrowth occurs [26]. Recurrence of clinical signs in this case 4 months after initial treatment supports this recommendation.

Historically, topical treatment has been considered contraindicated in cases with cribriform or sinus compromise, due to the potential for refractory seizures [16]. No significant complications were noted in two retrospective studies of dogs with cribriform plate defects managed with topical therapy [26, 29], but the impact of direct debridement and topical treatment of herniated brain tissue with clotrimazole is unknown. As a result, prophylactic intravenous antiepileptic therapy was administered during the procedure as per recommendations of the attending neurologist. Due to concern for inoculation of fungus into the encephalocele during debridement, a brief course of oral posaconazole also was added to the dog's treatment regimen. The dog in our case experienced recurrent seizures 3 years following the initial rhinoscopy procedure. It is unknown whether this is due to owner compliance, drug resistance, a delayed complication following the debridement and topical treatment, or again related to the patient's meningoencephalocele. The latter is considered most likely.

## 4. Conclusion

This case report describes successful treatment of sinonasal aspergillosis in a dog with a preexisting meningoencephalocele. Despite herniation of the olfactory bulb into the nasal cavity through a large cribriform plate defect resulting in exposure of the meninges, no complications were noted secondary to aggressive debridement of *Aspergillus fumigatus* plaques and repeated lavage and clotrimazole application as per standard protocols. Consistent with previous reports, diagnostic imaging and rhinoscopy were instrumental in disease diagnosis and case management. Recurrence of clinical signs 4 months after initial treatment due to residual disease highlights the importance of repeat rhinoscopy in limiting sequelae of ongoing colonization and achieving disease cure.

## Data Availability

All data relevant to the study are included in this article.
